# **“**Song of Life (SOL)**”** study protocol: a multicenter, randomized trial on the emotional, spiritual, and psychobiological effects of music therapy in palliative care

**DOI:** 10.1186/s12904-019-0397-6

**Published:** 2019-01-30

**Authors:** Marco Warth, Friederike Koehler, Martin Weber, Hubert J. Bardenheuer, Beate Ditzen, Jens Kessler

**Affiliations:** 1 Institute of Medical Psychology, Center for Psychosocial Medicine, University Hospital Heidelberg, Bergheimer Str. 20, 69115 Heidelberg, Germany; 2 Interdisciplinary Palliative Care Unit, III. Department of Medicine, University Medical Center of the Johannes Gutenberg University of Mainz, Mainz, Germany; 3 Center of Pain Therapy and Palliative Care Medicine, Department of Anesthesiology, University Hospital Heidelberg, Im Neuenheimer Feld 131, 69120 Heidelberg, Germany

**Keywords:** Music therapy, Palliative care, Cancer, Randomized controlled trial, End-of-life, Quality of life, Spiritual well-being, Distress, Ego-integrity, Cortisol, Stress, Heart rate variability

## Abstract

**Background:**

Although patients in palliative care commonly report high emotional and spiritual needs, effective psychosocial treatments based on high quality studies are rare. First research provides evidence for benefits of psychosocial interventions in advanced cancer care. To specifically address end-of-life care requirements, life review techniques and creative-arts based therapies offer a promising potential. Therefore, the present study protocol presents a randomized controlled trial on the effectiveness of a newly developed music therapy technique that is based on a biographically meaningful song (“Song of Life”; SOL).

**Methods:**

In a design with two parallel arms, 104 patients at two palliative care units will be randomly assigned to three sessions of either SOL (experimental group) or relaxation exercises (control group). Improvements in the psychological domain of quality of life will be the primary endpoint, while secondary outcomes encompass spiritual well-being, ego-integrity, overall quality of life, and distress. Additionally, caregivers will be asked to provide feedback about the treatment. Assessment of biopsychological stress markers and qualitative analysis of perceived strengths and weaknesses will complement data collection.

**Discussion:**

Based on the results of a previous pilot study, we dedicated considerable efforts to optimizing the intervention and selecting appropriate outcomes for the present trial. We are confident to have designed a methodologically rigorous study that will contribute to the evidence-base and help to develop the potential of psychosocial interventions in palliative care.

**Trial registration:**

German Clinical Trials Register (DRKS) – DRKS00015308 (date of registration: September 07th 2018).

## Background

How to preserve the psychosocial, spiritual and existential integrity of people facing an incurable disease is considered one of the main challenges of palliative care [1]. Although palliative care aims to support terminally ill patients and their relatives on a physical, psychosocial and spiritual level [2], effective treatments have mainly been developed and investigated with a bio-medical focus on pain or dyspnea, for instance [3]. Nevertheless, psychological distress (e.g., depression, anxiety or feelings of hopelessness) and spiritual concerns (e.g., regarding purpose or meaning in life) are highly prevalent in the terminal phases of a life-threatening disease [4, 5]. Thus, the recent advances of psychosocial interventions addressing these needs offer huge potential for providing holistic palliative care [4].

Interestingly, the link between psychological and biological mechanisms in terminal disease offers an encouraging psychoneuroimmunologcial perspective for psychosocial interventions [6]. On the one hand, the stress a patient experiences can increase through the diagnosis and course of a life-threatening illness like cancer, while on the other hand stress itself can also affect disease outcomes like tumor growth, progression and metastasis [7]. The human biological stress response is primarily adaptive and consists of an increased activation of the hypothalamic-pituitary-adrenal (HPA) and the autonomous nervous system (ANS). The resulting elevated levels of circulating glucocorticoids and catecholamines can suppress immunological activities and deteriorate symptoms, especially in chronical diseases like cancer [8]. Therefore, beyond pharmacological treatments, stress-reducing psychosocial interventions have been developed and shown to improve immune function [9, 10].

Most commonly, psychosocial treatments in palliative care are categorized into cognitive-behavioral therapy (CBT), mindfulness-based interventions, life review or meaning-centered interventions, and creative-arts based therapies [11]. Although CBT has shown to improve depression and quality of life in patients with advanced cancer [12], the treatment manuals mostly fail to consider the unique conditions of end-of-life care, such as rapid physical declines or limited life expectancy [13]. Mindfulness-based interventions focus on the cultivation and practice of mindfulness (i.e., purposeful, nonjudgmental, moment-to-moment awareness) [14]. In cancer care, research has proliferated over the past decade with evidence on improvements in psychological distress and sleep disturbances as well as markers of immune function and autonomous nervous system activity [15]. Despite its potential for palliative care settings, a recent review on short-term mindfulness interventions [16] identified only a limited number of clinical trials with low quality of evidence.

To specifically address the psychological and existential distress of terminally ill patients, life review interventions such as dignity therapy [17, 18], the structured life review [19], or meaning-centered therapy [20] have recently emerged. According to developmental psychologist Erik Erikson, the central themes of the final stages of life can be outlined as generativity (i.e., producing something lasting of oneself) and ego-integrity (i.e., a retrospective sense of acceptance and meaning of one’s life) [21]. The life review techniques approach generativity and ego-integrity of terminally ill patients through creating an intellectual or physical legacy, e.g., a written transcript on the patient’s life history and hopes for their loved ones or a biographical photo book [13]. Although patients, relatives and professionals generally report high satisfaction and an improved end-of-life experience through life review interventions [17, 22], empirical findings remain inconclusive concerning reproducible effects on validated quality of life measures [23, 24]. Cumulative evidence is offered by a recent meta-analysis demonstrating moderate effects on spiritual well-being, general distress and quality of life, but with a high risk of bias [25].

Arts and music in creative-arts based therapies can also be used as a means to facilitate feelings of meaning and a psycho-spiritual integration of life experiences in terminally ill patients and caregivers [26, 27]. The use of music therapy in multidisciplinary palliative care dates back to the 1970s, when the Canadian music therapist Susan Munro as a pioneer systematically described her work with palliative patients on a physical, psychological, social, and spiritual level [28]. Nowadays, music therapy has become a widely implemented and explicitly recommended complementary therapy in palliative treatment [29]. Music therapy is generally defined as “a systematic process of intervention wherein the therapist helps the client to promote health, using music experiences and the relationships that develop through them as dynamic forces of change” [30]. In clinical palliative care practice, music therapy aims to improve quality of life through the relief of physical symptoms and psychological difficulties by offering comfort, facilitating communication and spiritual experiences [31].

Music therapy includes active techniques (the patient is actively involved in the production or reproduction of music) as well as receptive techniques (the patient listens to live or prerecorded music). Techniques in palliative care have been categorized as [32]: receptive (e.g., imagery), creative (e.g., songwriting), recreative (e.g., instrument playing) and combined (e.g., musical life review). The latter area indicates a possible synthesis of life review techniques with the help of music, especially concerning the improvement of both psychological and existential distress [32]. Despite a lack of empirical research, Sato [33] proposes a first experience-based Musical Life Review Model describing the stimuli, themes, responses, evaluation and therapeutic outcomes of this process.

While clinical practice emphasizes the benefits of music therapy in end-of-life care [28], there is still insufficient research to reliably conclude a favorable effect due to a lack of empirical investigations in both quantity and quality. Most palliative care referrals for music therapy focus on pain and quality of life with a low level of evidence [34]. For instance, music therapy has been frequently associated with pain reduction [35, 36] and enhancement of physical comfort [37, 38]. With regards to quality of life, patients receiving music therapy often report higher psychophysiological well-being [39] and subjective well-being [40] as well as improvements in emotional distress [41], anxiety and mood [36, 42, 43]. Additionally, single studies report an enhancement of spirituality [44] and lowering of salivary cortisol levels [43] after music therapy. Even family caregivers and staff members attribute music therapy an important role in the end-of-life experience [45]. However, the abovementioned studies reveal a high risk of bias (e.g. no randomization or allocation concealment, selective reporting). Thus, methodologically rigorous studies on clearly defined music therapy interventions with common outcome measures are urgently needed.

Overall, to contribute to this important but underrepresented topic of psychosocial interventions in palliative care, the general aim of the present study is to evaluate the effectiveness of the newly developed music therapy technique “Song of Life” in terminally ill patients with regard to the psychological and spiritual domain of quality of life. Based on a biographically meaningful song, SOL integrates aspects of music therapy, musical life review, and dignity therapy. In the present study, we will compare the effects of SOL to the ones of a control group which will participate in live guided relaxation exercises.

## Methods / design

### Participants and setting

This multicenter study will be conducted in parallel at two sites: The University Palliative Care Unit at the St. Vincentius Hospital in Heidelberg, Germany, and the Interdisciplinary Palliative Care Unit at the University Medical Center in Mainz, Germany. Patients admitted to these units will be screened for eligibility based on their medical record and information from personal contact. Patients are eligible for participation if they meet the following criteria: a) Palliative care according to OPS 8–982/OPS 8.98e or estimated life expectancy < 12 months, b) age ≥ 18 years, c) ability to provide informed consent, d) sufficient understanding of German language, d) clinical estimation of life expectancy > 1 week, e) no cognitive or auditory impairments (including severe psychiatric symptoms). Criteria d) and e) will be assessed by the treating physician.

Patients will be asked to name a family member, friend or other close person who is currently involved in providing assistance (subsequently called *caregiver*) whom we may contact for a caregiver evaluation of the intervention. Caregivers will be included in this study if they a) are 18 years or older, and b) show sufficient understanding of German language. All participants will be informed by the outcome assessor and need to sign consent before participation.

### Study design

**Fig. 1:**
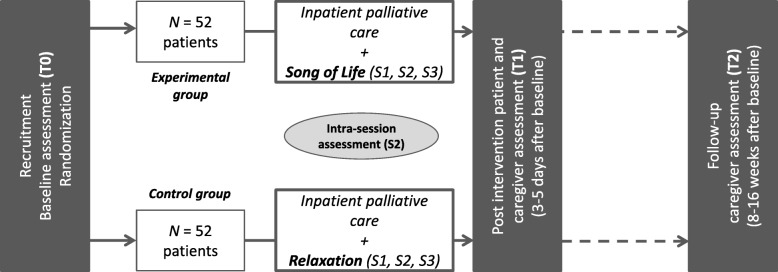
Study design. Notes: S = therapeutic session, T = time of assessment

The study utilizes a prospective, randomized controlled design with two parallel arms. Patients will be randomly assigned to three sessions of either music therapy (EG) or relaxation exercises (CG). We will not give information about which of the two alternative treatments is the experimental and which is the control condition. Hence, patients will be blinded with regard to the study hypotheses. It will not be possible to blind the therapists and outcome assessors, but all data will be assessed by persons different than the study therapists (i.e. study nurse, doctoral student). We will use a computer-based block randomization procedure with a block size of 8. A random sequence for group assignment will be created before the first patient is randomized. Based on this sequence, we will use the SNOSE-method [46] to conceal allocation. We expect benefits and improvements in both treatment groups with a superiority of the SOL intervention. The study design is summarized in Fig. 1.

### Patient procedures

**Fig. 2:**
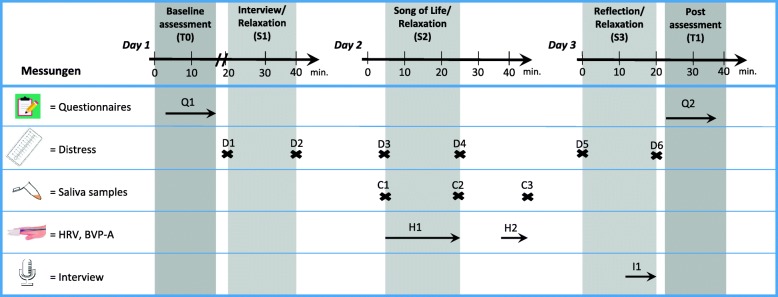
Patient assessments. Notes: HRV = heart rate variability, BVP-A = blood volume pulse amplitude, S = therapeutic session, T = overall timing of assessment, Q = timing of questionnaire assessment, D = timing of distress assessment, C = timing of cortisol /α-amylase assessment, H = timing of cardiovascular recordings, I = timing of interview

Eligible patients will be informed about the study goals, procedures, data protection plan, risks, and benefits. Each patient agreeing to participate and providing informed consent will undergo a baseline assessment (T0) including questionnaire data on different quality of life domains. Afterwards, a study assistant will open a serially numbered, sealed envelope containing information about group assignment. In each group, appointments will be made for three sessions of either music therapy (EG) or relaxation (CG), ideally on consecutive afternoons. Each of the three sessions is enclosed by a pre-to-post assessment of momentary distress. For both groups, the second session contains a psychobiological intra-session assessment. Post intervention data (T1) will be collected after completion of the third session including all variables assessed at baseline (T0) plus a retrospective evaluation of treatment quality. Sessions and assessments will last for approximately 45 min each day. Figure 2 depicts an overview of the timing of assessments. The interventions and outcomes will be described in detail in the following paragraphs.

### Caregiver procedures

If the caregiver has given consent to participate, we will contact the caregiver proposed by the patient either in person or by phone after completion of the last session. The reason for the inclusion of caregiver evaluations in the study design is the possibility to gather indirect information on the endurance of effects. Caregivers will be provided with all relevant information concerning their study participation and asked to sign the consent form. Subsequently, they will participate in a brief structured interview on their retrospective evaluation of treatment quality (T1). We will ask for permission to contact the caregiver again after 8 weeks (T2). On this follow-up assessment, caregivers will answer the same questions as at T1 in a telephone interview. If the caregiver expresses intense distress or unease, we will offer to postpone the appointment. To be included in the analysis, follow-up assessments (T2) need to be completed within 16 weeks after T1.

### Interventions

The intervention in the EG is a newly developed music therapy technique specifically designed to address psycho-spiritual needs in an end-of-life care context by using a creative-arts based technique (“Song of Life”). The core element of SOL is a biographically meaningful song, based on the “Song of kin” music therapy technique of neonatal care [47, 48]. The song of kin is a parent-selected song performed as a lullaby and synchronized with the infant’s vital signs [49]. The SOL-technique has shown to be feasible and accepted in palliative care in a recent pilot study [50]. We expect each of the three therapeutic sessions (S) to last between 20 and 35 min:

- S1 (Interview): The first session comprises an interview including several biographical questions with a focus on the musical background of the person. The last items specifically aim to identify the patient’s SOL, which is a song that has a high biographical relevance and the potential to arouse strong emotional reactions [47, 48, 51]. Our pilot study [51] showed that such a song is often associated with social bounds (e.g. romantic partner, children, parents), biographic events (e.g. marriage, child’s birth), or certain places (e.g. birthplace, vacation). The first session ends with the offer to perform a brief music therapy relaxation exercise using the monochord and vocal improvisation.
- S2 (“Song of Life”): The second session will be carried out the next afternoon to give the therapist time to prepare the patient’s SOL. The therapist uses the guitar/piano and her voice and first initiates a brief relaxation exercise accompanied by gentle sounds on the instrument in the musical key of the following song. If possible, the musical beat entrains to the patients’ breathing cycle and the therapist gradually reduces the meter [52]. If applicable, the therapist can include quotations of important sentences from the interview in S1. The music afterwards turns into a life performance of the SOL in a lullaby style. This requires the therapist to translate the original version into a slow 3/4-rhythm or 6/8-rhythm. The song performance is faded out with a diminuendo of the hummed song chorus.
- S3 (“Reflection”): The live performance will be recorded and edited after S2 by the therapist or study assistant, and will be handed over to the patient in S3. Therapist and patient can complete the intervention by listening together to the recording and reflect on the statements and messages. After this initial phase of free conversation, the therapist will guide the talk according to themes of a semi-structured qualitative interview on the patient’s perceptions, attitudes, and beliefs towards the strengths and weaknesses of the SOL intervention. At the end, the therapist can offer to again play a brief music therapy relaxation on the monochord.

The control group intervention consists of three sessions of relaxation exercises, carried out by the music therapists involved in this study. A professional mindfulness trainer created three standardized, consecutive exercises for supine positions for the purpose of this study. Each exercise will last for approximately 20 min. Elements of these sessions are guided relaxation, focused breathing, and mindfulness. The techniques will not contain musical elements and will not target biographical or spiritual themes. Therapists at both study sites have been trained in conducting the SOL and relaxation treatment before the onset of the intervention period.

As study participation is voluntary, the patient can cancel the intervention at any given point (e.g., due to worsening physical states). In this case, it remains the patient’s decision whether the data collected up to this point should be deleted. If the patient consents, self-report data on the primary and secondary outcomes (see below) will be collected together with reasons for drop-out.

### Outcomes

Previous psychosocial research often used generic quality of life measures, palliative performance, or the concept of dignity as the primary outcome and faced difficulties in identifying significant effects on these constructs (which were operationalized including items on functional or physical state) [53]. The present SOL intervention is assumed to work on an emotional and spiritual level. Therefore, improvement in the psychological domain of quality of life (a1) is chosen as the primary patient endpoint, while the secondary outcomes encompass spiritual well-being (b1), ego-integrity (b2), general quality of life (b3), distress (b4), and the retrospective evaluation of the interventional benefits (b5). To gather additional information about the mechanisms underlying a potential effect, we will assess psychobiological markers of stress (c1) as well as subjective beliefs and attitudes towards the treatments in a qualitative interview (c2). As stated above, the family caregiver’s perspective will be captured both after the intervention and 8–16 weeks later (d1). A study assistant and a doctoral student will carry out all outcome assessment, including questionnaires, photoplethysmography, saliva samples, and caregiver interviews.

Table 1 gives an overview of the study outcomes for patients and family caregivers. The timing of assessments can be found in Fig. 2.

**Table 1:** Study outcomes

	Primary outcome	Secondary outcomes	Exploratory analyses
Patient	• a1: Quality of life – psychological (MQOL-R, 4 items, T0-T1)	• b1: Spiritual well-being – meaning/peace (FACIT-Sp, 8 items, T0-T1) • b2: Ego-integrity (Brief Measure of Generativity and Ego-Integrity, 5 items, T0-T1) • b3: Quality of life - global (MQOL-R, 1 item, T0-T1) • b4: Distress (NCCN Distress Thermometer, 1 item, pre-post each session) • b5: Retrospective evaluation (Feedback Questionnaire, 8 Items, T1 only)	• c1: Biomarkers of stress: ▪ HPA-axis (cortisol) ▪ Sympathetic nervous system (salivary α-amylase, peripheral blood flow) ▪ Parasympathetic nervous system (HRV) • c2: Strengths and weaknesses of the intervention; Beliefs about working mechanisms (qualitative interview)
Caregiver		• d1: Retrospective evaluation (Feedback Questionnaire, 12 items, T1-T2)	

*FACIT-Sp* Functional Assessment of Chronic Illness Therapy – Spiritual Well-being, *MQOL-R* McGill Quality of Life Questionnaire Revised, *NCCN* National Comprehensive Cancer Network, *HPA* hypothalamic-pituitary-adrenal, *HRV* heart rate variability, *T0* baseline assessment, *T1* post intervention assessment

#### Primary outcome

1) The *psychological subscale* of the *McGill Quality of Life Questionnaire - Revised (MQOL-R)* [54, 55] will be used to measure changes in emotional well-being at the end of life from T0 (pre intervention) to T1 (post intervention). The MQOL was originally developed in 1996 for patients with life-threatening diseases and has recently been shortened and revised [56]. The current version consists of 14 items and four subscales (physical, psychological, existential, social), which all showed acceptable internal consistency. Confirmatory factor analysis provided support for the assumed measurement structure [56]. The psychological subscale contains 4 items on 11-point scales asking for issues related to depression and anxiety in the past 2 days.

#### Secondary outcomes

1) We will assess changes on the 8-item *meaning/peace-scale* of the *Functional Assessment of Chronic Illness Therapy - Spiritual Well-Being (FACIT-Sp)* to measure non-religious aspects of spiritual well-being. The FACIT-Sp is an extended version of the *Functional Assessment of Cancer Therapy – General (FACT-G)* and is commonly used in cancer and palliative care research. Adequate psychometric properties were identified for both the FACT-G [57] and the FACIT-Sp [58]. We will reduce the time frame in the items from seven to three days.

2) According to Erikson’s stages of psychosocial development [21], to gather acceptance and a sense of meaning regarding one’s past life is an important developmental task in the last stage of life. We will measure progress in this adaption process using the 5-item *ego-integrity subscale* of the *Brief Measure of Generativity and Ego-Integrity* [59]. The questionnaire’s factor structure has been confirmed in a validation study.

3) The *MQOL-R’s overall quality of life* single-item scale [56] will be used to briefly measure treatment effects on general well-being. The item asks for overall quality of life during the last 48 h from *very bad (0)* to *excellent (10).* The abovementioned secondary outcomes b1 to b3 will all be assessed at baseline (T0) and after completion of session 3 (T1).

4) Additionally, we will utilize a modified version of the *NCCN Distress Thermometer* [60] before and after each session to monitor changes in momentary distress (D1-D6, Fig. 2). The single-item scale has a response range from *no distress (0)* to *extreme distress (10)*. The original version is a commonly used screening instrument in cancer care and research asking for distress in the past week, while an existing modified and more change-sensitive version asking for current distress will be used in the present study [61].

5) Post-intervention assessment (T1) will be complemented by a shortened and modified version of the retrospective *Feedback Questionnaire*, previously used in dignity therapy research [17]. We will use eight items on a 5-point scale covering aspects of the patient’s subjective perception of treatment effects. For each item, patients can state reasons for their rating in an open format.

#### Exploratory analyses

1) In order to understand more about the mechanisms underlying a potential treatment effect, we will collect and record psychobiological data on stress reactivity during session 2 (which contains the performance of the SOL). Due to the paucity of research regarding the effects of psychosocial interventions on biological stress markers in palliative care, we decided to rather explore trajectories in a mechanistic approach than to state explicit hypotheses. The psychobiological assessments in this study aim at monitoring states and changes in the following regulatory systems:

- Hypothalamic-pituitary-adrenal (HPA) system via salivary cortisol
- Sympathetic nervous system (SNS) via salivary α-amylase and peripheral blood flow
- Parasympathetic nervous system (PNS) via heart rate variability (HRV)

It is important to note that these stress markers will be collected non-invasively by means of saliva samples (cortisol, α-amylase) and photoplethysmography (blood flow, HRV) aiming to minimize patient burden. The timing of assessments is depicted in Fig. 2. Saliva samples will be collected at three measurement times during session 2 (C1-C3, Fig. 2) for the analysis of cortisol and α-amylase. These therapy sessions and data collection will preferably take place at standard times during the day (between 2 pm and 6 pm).

Cortisol is the end-product of a cascade of neuroendocrine responses to physically or psychologically challenging situations. As free cortisol concentration in saliva reaches its peak approximately 20 min after central activation of the HPA axis in response to a stimulus [62], the measurement points C1-C3 correspond to:  20 min. before Baseline (C1), pre intervention (C2), and post intervention (C3). The Cortisol Salivette® (Sarstedt, Nümbrecht, Germany) will be used for saliva sampling. Patients are asked to chew on a small synthetic swab for 1 min for each saliva collection. Swabs will be placed in sterile plastic centrifugation tubes. All samples will be stored at − 80 °C at the laboratory of the Institute of Medical Psychology (IMP), where they will later be centrifuged and analyzed using standard commercially available ELISA.

We will additionally calculate concentrations of salivary α-amylase (sAA) from the same saliva samples as a measure of SNS activity. sAA is an enzyme mainly secreted by the parotid glands in response to adrenergic innervation and has thus been studied as a proxy for the sympathetic modulation of acute responses to stress [63–65]. Short-term increases in sAA release were observed in psychologically challenging situations [66] and in response to pharmacological stimulation of adrenergic receptors [67].

We acknowledge the dependence of the endocrine biomarkers on exogenous glucocorticoids, chemotherapy, and opioid administration [68, 69]. The majority of patients in palliative care requires medication intake from multiple of these classes. Our interest, however, lies not in the absolute cortisol levels, but in the reactivity to the interventions provided, and hence, in the exploration of intra-individual change over time.

Additionally, we will use photoplethysmography for non-invasive, continuous recordings of participants’ autonomic response before, after, and during the interventions. A Blood Volume Pulse Sensor (biosignalplux, Lisbon, Portugal) will be placed on the index fingertip of the non-dominant hand, detecting relative changes in peripheral circulation [70]. The time point of the R-wave is used to extract inter-beat-intervals (IBI) between successive heartbeats in milliseconds. HRV parameters can be derived for consecutive time segments of 5 min duration [71]. High-frequent HRV is in indicator for vagal modulation of cardiac outflow [72–75] and has shown to correspond to self-ratings of relaxation and well-being in response to psychosocial interventions in palliative care [76, 77].

The amount of peripheral blood flow in the fingertips’ capillaries is predominantly subject to adrenergic innervation by the SNS, leading to tonic vasoconstriction. An increase in blood volume pulse amplitude (BVP-A) is therefore associated with a reduction in sympathetic arousal. Thus, the mean BVP-A of each 5-min segment will be considered an inversely related index of vascular sympathetic tone [70, 78–80]. We will continuously record the first 20 min of session 2 (H1) which includes the live-performance of the chosen SOL, and another 5-min segment 20 min later (H2), in parallel to saliva sample C3 (Fig. 2).

2) After an individual reflection conversation about the SOL and listening to the music, the study therapists will guide the second part of session 3 on the basis of a semi-structured interview (I1, Fig. 2). Open questions will address perceived strengths and weaknesses of the treatment, possible adverse effects as well as subjective explanations for potential effects. Our goal is to gain additional insights about the underlying mechanisms of the SOL intervention. The qualitative interview will last for approximately 10 min and will be carried out and recorded only in the EG.

#### Caregiver evaluation

The retrospective patient Feedback Questionnaire exists in a family caregiver version, offering the opportunity to explore the duration of effects from a caregiver perspective [1, 81]. In case of prior consent, the caregivers’ evaluation of treatment quality will therefore be assessed after the treatment (T1) and 8–16 weeks later (T2).

### Data monitoring and management

Data collection and conduction of trial will be monitored by the principal investigators. Adverse or unintended effects of interventions or conduct will be reported and managed in standard palliative care. In order to protect confidentiality, all data and biomaterial will be collected and stored for 10 years in a pseudonymized form at the Center for Psychosocial Medicine at University Hospital Heidelberg. Only the principal investigators will have access to the assignment key. No third parties are allowed inspection of personal data. After the end of the research project, data will be published in online repositories in an anonymized form.

### Data analysis

Data will primarily be analyzed within an intention-to-treat approach. Results will be tested for robustness in comparison to a per-protocol-analysis (sensitivity analyses). Primary hypotheses tests will be conducted with analyses of co-variance (ANCOVA). The study design contains two independent variables to predict variance in the primary outcome: 2 groups (between-subjects factor) * 2 measurement times (within-subjects factor). In order to increase statistical power, the ANCOVA models will treat pre-intervention baseline data as a covariate and group assignment as a fixed factor [82]. The following primary hypotheses will be tested in a confirmatory ANCOVA with a type-I error probability of α = 0.05: There is a significant effect of group assignment on psychological quality of life after the intervention (while controlling for baseline differences). Group differences in the changes in secondary outcomes will be tested accordingly without adjustment for multiple testing, due to the a priori distinction between primary and secondary outcomes and the likelihood of the outcomes to correlate.

In addition to statistical significance, we will report descriptive statistics and effect size estimates. Biomarker analysis will be conducted by use of multilevel modeling, as repeated observations are nested within participants, and experience from previous research revealed substantial heterogeneity in slopes and trajectories among severely ill patients [77, 83]. Multilevel modeling can be further used for subgroup analysis (e.g. gender differences), for moderator analysis (e.g. the influence of patient distress), and to account for confounding variables (e.g. baseline differences, sex, age). Qualitative data from session S3 will be recorded, transcribed, coded, and analyzed in accordance with Mayring’s approach to qualitative content analysis [84].

### Sample size

Sample size calculations for this study are adjusted for intervention effects on psychological quality of life (i.e. the primary outcome). A systematic review [85] suggests that medium-sized effects are most likely to be expected for the impact of music therapy on psychosocial outcomes in palliative care. Our own studies support this assumption finding effect sizes varying between *d* = 0.35 and *d* = 0.80 for generic quality-of-life measures [40]. Recent articles [82, 86] on sample size calculations emphasize the superiority of ANCOVA models for analyzing the results of RCTs over the repeated-measures ANOVA approach, as the inclusion of pretest values as a covariate increases statistical power. Assuming statistical power of (1- α) = 0.8, type-I error probability of α = 0.05, and a correlation between covariate and outcome of ρ = 0.6, a total sample size of *N* = 84 is required to detect medium sized effects of *d* = .50 between groups [86]. With an expected dropout rate of approximately 25%, *N* = 104 participants need to be recruited for this study.

## Discussion

We present the protocol of a randomized controlled trial on the emotional, spiritual, and biological effects of the “Song of Life” intervention for patients receiving palliative care. Our decisions for the chosen research methods are the result of intensive, critical and interdisciplinary discourse between researchers and clinicians from psychology, medicine, and music therapy. Although we are confident to have designed a robust and sound approach to study the effects of the SOL intervention, we would like to discuss some potential methodological pitfalls.

First, the design of an appropriate control intervention is crucial for the interpretation of effects. Most studies on psychosocial interventions in palliative care compare the actual treatment to a “standard care” or “treatment as usual” group. While it is more likely to identify significant effects in comparison to a group that does not receive any additional treatment, these effects cannot be causally attributed to the intervention, as attentional, expectancy and other biases can play a role. Moreover, it can be argued that it is unethical to include terminally ill patients in a study and then withhold a potentially effective treatment. In a previous trial, we therefore compared a music therapy relaxation exercise to a prerecorded verbal relaxation exercise [87]. While this was a methodological improvement over many studies in the field, we still could not rule out the possibility that the personal attendance to the patient in the experimental group contributed to the observed effects. Hence, we decided to use an active and effective control intervention carried out by trained therapists in the present study. We did not include a third “standard care” arm as this could raise ethical concerns and would have further increased the required number of participants.

We are aware that direct comparison to an active control treatment will diminish the magnitude of observed effects because we expect the relaxation exercises to lead to substantial improvements as well. This could become particularly challenging, as our pilot study showed only medium-sized effects from pre to post for the unmodified version of the SOL [50]. However, our response was not to dilute the study design, but to put effort in optimizing the treatment and selecting appropriate outcomes. We would like to encourage all readers to interpret the upcoming results of the study accordingly: Non-significant findings would not mean “not effective” but rather “not more effective than relaxation exercises”, while significant findings in the expected direction would mean “even more effective than relaxation exercises”.

We believe the major strengths of this study to be the innovative intervention that was specifically designed to address the needs of patients nearing the end of life and the comprehensive assessment plan including emotional, spiritual, and biological outcomes. Moreover, the comparison to an active control treatment is unique in the study of music therapy in palliative care and will significantly reduce the likelihood of attention and expectation bias [88]. Finally, we would like to emphasize that the present paper is an a priori study protocol, which means that the primary and secondary outcomes were registered and made public before the first patient was randomized which will diminish the risk of “*p*-hacking” and reporting bias.

The present study will help to understand the potential of psychosocial interventions for terminally ill patients and will particularly contribute to the evidence base of creative-arts based therapies in palliative care.

## Abbreviations

AN(C)OVA: analysis of (co-)variance
ANS: autonomous nervous system
BVP: blood volume pulse
CBT: cognitive-behavioral therapy
CG: control group
EG: experimental group
ELISA: enzyme-linked immunosorbent assay
FACIT-Sp: Functional Assessment of Chronic Illness Therapy – Spiritual Well-Being
FACT-G: Functional Assessment of Cancer Therapy – General
HPA: hypothalamic-pituitary-adrenal
HRV: heart rate variability
IBI: inter-beat-interval
ICD: International Statistical Classification of Diseases and Related Health Problems
MQOL-R: McGill Quality of Life Questionnaire Revised
NCCN: National Comprehensive Cancer Network
OPS: Operationen- und Prozedurenschlüssel (German procedure classification)
PNS: parasympathetic nervous system
RCT: randomized controlled trial
S: session
sAA: salivary α-amylase
SNOSE: sequentially numbered, opaque sealed envelopes
SNS: sympathetic nervous system
SOL: Song of Life
